# Out-of-pocket payments and catastrophic household expenditure to access essential surgery in Malawi - A cross-sectional patient survey

**DOI:** 10.1016/j.amsu.2019.06.003

**Published:** 2019-06-11

**Authors:** Leon Bijlmakers, Maike Wientjes, Gerald Mwapasa, Dennis Cornelissen, Eric Borgstein, Henk Broekhuizen, Ruairi Brugha, Jakub Gajewski

**Affiliations:** a Radboud University Medical Centre Netherlands, Geert Grooteplein Zuid 10, 6525, GA, Nijmegen, the Netherlands; b College of Medicine, Malawi, Mahatma Gandhi, Blantyre, Malawi; c Royal College of Surgeons in Ireland, 123 St Stephens Green, Dublin 2, Ireland

**Keywords:** Surgery, Household cost, Out-of-pocket, Catastrophic expenditure, Malawi

## Abstract

**Background:**

Having to pay out-of-pocket for health care can be prohibitive and even cause financial catastrophe for patients, especially those with low and irregular incomes. Health services at Government-owned hospitals in Malawi are provided free of charge but patients do incur costs when they access facilities and some of them forego income. This research paper presents findings on the direct and indirect expenditure incurred by patients who underwent hernia surgery at district and central hospitals in Malawi. It reports the main cost drivers, how costs relate to patients’ household incomes, the financial burden of undergoing surgery and the extent to which hernia patients had recovered and restored their capacity to work and earn an income.

**Materials and methods:**

Using a cross-sectional study design, surveys were held with patients who had undergone hernia surgery in four district and two central hospitals in Malawi. Interviews were conducted by surgically trained clinical officers, trained in survey administration, and included, inter alia, questions about patients’ hospital stay, the direct and indirect cost they incurred in accessing surgery, and how they financed the expenditure. Follow-up interviews by telephone were held 8–10 weeks after discharge.

**Results:**

The sample included 137 patients from district and 86 patients from central hospitals. The main direct cost drivers were transport and food & groceries. More than three quarters of patients who had their surgery at a district hospital incurred indirect costs, because of income lost due to hospital admission, compared with just over a third among central hospital patients. Median reported income losses were US$ 90 and US$ 71, respectively. Catastrophic expenditure for surgery occurred in 94% of district and 87% of central hospital patients. When indirect costs are added to the out-of-pocket expenditure, it constituted more than 10% of the monthly per capita income for 97% and 90% of the district and central hospital patients, respectively.

**Conclusion:**

Out-of-pocket household expenditure associated with essential surgery in Malawi is high and in many instances catastrophic, putting households, especially those who are already poor, at risk of further impoverishment. The much needed scaling-up of surgical services in rural areas of Malawi needs to be accompanied by financial risk protection measures.

## Highlights

• Out-of-pocket household expenditure associated with essential surgery in Malawi is high and in many instances catastrophic.
• Income foregone because of hospital admission for surgery often exceeds patients' direct out-of-pocket expenditure.
• Coping mechanisms, such as taking out loans or selling assets, are fairly common, indicating a risk of impoverishment.
• Scaling-up of surgical services needs to be accompanied by financial risk protection measures.

## 1 Introduction

Whereas approximately 30 percent of the global burden of disease is surgical, nearly 5 billion people worldwide are without access to safe, affordable and timely surgical and anaesthesia care [1]. Among those who do access care, an estimated 33 million every year face financial hardship from the direct costs of surgery, with another 48 million incurring financial catastrophe from the non-medical costs of transportation, food and lodging necessary to obtain surgery [2]. The Lancet Commission on Global Surgery developed six core surgical indicators, to monitor progress towards universal access to surgical care at national and global levels. Two of these relate to the impact of surgical care: protection against impoverishing expenditure and catastrophic expenditure [3]. Country-level estimates of the financial burden of surgery do exist, but few are based on empirical studies. Patient costs are considered one of the main barriers for patients in accessing surgical services [4]. Out-of-pocket expenditure may involve direct payments – for consultation, medicines, or investigations – or indirect payments by patients and their relatives for transportation, lodging and food. Since patients travel long distances to a referral hospital, the time involved as well as the cost of transportation – in case the referring hospital does not provide ambulance services free of charge – can be reasons not to comply with the advice to seek (surgical) care at a referral hospital [5]. In case of a life threatening condition that requires emergency evacuation any delay can be fatal. For those who do seek surgical care, their out-of-pocket payments can be catastrophic, with possible impoverishing effects on their households.

It has been well documented that households in low- and middle-income countries are being pushed into poverty when confronted with substantial medical expenses that are not covered by any form of health insurance [6,7]. In such situations, patients pay substantial amounts of money out-of-pocket, with some taking out loans to cover the expense [8,9], with further impacts if patients experience loss of income due to their illness. The poverty deepening impact of direct out-of-pocket payments for medical care, combined with opportunity costs due to loss of income, particularly affects people below the poverty line [[10], [11], [12], [13], [14]].

Catastrophic health expenditure is defined as direct out-of-pocket payments for accessing health services that exceed 10% of the total monthly per capita income [15]. In their modelling study, Shrime et al. [2] made global estimates of catastrophic expenditure to pay for surgery, showing that 3.7 billion people worldwide (about half the global population) are at risk of catastrophic expenditure for surgery. Each year, surgical conditions drive 81.3 million people worldwide to financial catastrophe, of which 32.8 million is from the costs of surgery alone and 48.5 million from associated non-medical costs. In a follow-up study, the same authors provided country-level estimates for a set of financial risk metrics related to surgery [16]. For Malawi they found the probability of catastrophic expenditure, if surgery is required, to be 57% for the country's population on average, but with clear differences between income groups, varying from 27% for the richest to 88% for the poorest income quintile. The probability of impoverishment at the national poverty line if surgery is required was estimated at 59% for the population on average, ranging from 1% for the richest to 100% for the two poorest income quintiles. Impoverishing expenditure for surgical care is defined as direct out-of-pocket payments for surgical and anaesthesia care which drive people below the poverty threshold [15]. Prior to the work by Shrime et al., the World Bank estimated that 92% of the population in Malawi were at risk of impoverishing health expenditure for surgical care [15].

The objective of this study was to measure the direct and indirect cost incurred by patients who underwent essential surgery at district and central hospitals in Malawi. The study established the main cost drivers, how costs relate to the patients’ household incomes, the financial burden of undergoing hernia surgery, the occurrence of catastrophic health expenditure, and the extent to which surgical patients had recovered and restored their capacity to work and generate income.

### 1.1 Study setting

Malawi has a population of 18 million, of which 84% live in rural areas [17]. With a Human Development Index of 0.477 in 2017, Malawi ranked 171th in the world (out of 189 countries), below the average of 0.504 for countries in the low human development group and below the average of 0.537 for all countries in sub-Saharan Africa combined [18]. The poverty headcount ratio at the national poverty line indicates that 51.5% of Malawi's population in 2016 lived below the poverty line (income of US$ 1.90 per capita per day) [17].

The country has a network of 24 government-owned district hospitals and 23 faith-based hospitals that provide surgical services in rural areas, where almost all surgery is undertaken by non-physician clinicians, locally called clinical officers [19]. Four of Malawi's cities each have a central hospital, where specialist surgeons are active. Four district hospitals (Mangochi, Mwanza, Nkhotakota and Nsanje) and two central hospitals (Kamuzu central hospital in Lilongwe and Queen Elizabeth central hospital in Blantyre) were selected for the present study.

## 2 Methods

Using a cross-sectional study design and based on a pre-tested structured questionnaire, interviews were conducted with patients who had undergone hernia surgery. Hernia repairs are among the most commonly performed surgical procedures performed in district and central hospitals in Malawi [19]. Clinical officers, who had received prior interviewer training, conducted the interviews in Chichewa, the local language. Interviewees were assured of the confidentiality of the process and their right to withdraw from the interview at any moment. Informed consent was obtained from all participants. Patients were interviewed one day before they were discharged from the hospital or on the day of discharge. The questionnaire covered health service utilisation prior to surgery, household composition, household income in the month prior to surgery, the types of expenditure that patients and their accompanying relatives had incurred to access surgical care, and the mode of financing. It also covered income foregone as a result of their hospital admission, where applicable. The interviewers entered the responses in an electronic (MS Excel-based) template before onward transmission of the data to the research coordinator (GM) for quality control. All interviews were held between May 2015 and June 2016.

Post-discharge follow-up interviews by telephone were held with study participants who had their surgery at a district hospital eight to ten weeks after they were discharged. These follow-up interviews (by GM) served primarily to establish whether the patients had recovered from surgery (the results have been are reported elsewhere [20]). They also asked if the respondents had experienced any financial burden post-operatively and whether they had regained their capacity to work and generate income, where applicable.

Statistical analysis was performed using SPSS Statistics 22. Since the expenditure data were not normally distributed, median values were calculated and, where appropriate, the inter-quartile range (IQR), by subtracting the 1st quartile from the 3rd quartile (IQR = Q_3_-Q_1_). Mann-Whitney U-tests served to examine differences between different categories: elective versus emergency hernia cases; patients operated in district hospitals versus those operated in central hospitals; and household income quintiles. All obtained costs were initially expressed in Malawian Kwacha (MWK), and later converted into US dollars (US$), using the exchange rate on 31st December 2015 of MWK 664.7 to US$ 1 [21].

The study was reviewed and approved by the University of Malawi College of Medicine Research Ethics Committee (ref no: P.03/12/1188). The work presented here is reported in line with the STROCSS criteria [22].

## 3 Results

A total of 137 patients from the four district hospitals were included in the study, with average age 46 (range 12–87), 82% males; and 86 patients from two central hospitals, average age 44 (range 19–93), 81% males, of which 26 (30%) had been referred by a lower-level health facility. Table A.5 in the Appendix shows the distribution of the interviewed patients over the six facilities. The case mix differed between district and central hospitals, with 6% and 29% emergency cases, respectively. Inguinal hernia repair was the most common procedure (170 cases for both types of hospitals combined; 76%), followed by epigastric (24 cases), (supra-)umbilical (9), incisional (8) and femoral hernia (3) repairs (missing information for 9 cases). The median duration of stay at district hospitals (pre- and postoperative periods combined) was 4.0 days, with an inter-quartile range (IQR) of 3.0. Patients at central hospitals had shorter stays: median 1.6 days (IQR = 1.8).

A minority of patients had arrived at the hospital by ambulance: 4% of the patients who had their surgery at a district hospital (6 cases), and 22% of those treated at a central hospital (19 cases). Most patients incurred costs for transport (fuel or bus services): 84% at both types of hospitals (Table 1). This includes the cost of transport for accompanying guardians, where applicable. The median expenditure on transport for patients treated at a district hospital was higher than for those who had their surgery done at a central hospital (MWK 2,700, the equivalent of US$ 4.06, versus MWK 1200 or US$ 1.81; p < 0.01).

**Table 1:** Transport expenditure incurred by patients and their guardians at district and central hospitals (in US$), emergency versus elective cases.

District hospitals	Emergency cases (n = 8)	Elective cases (n = 121)	Unknown (n = 8)	Total (N_1_ = 137)
Patients who incurred transport cost	8 (100%)	102 (84%)	5 (63%)	115 (84%)
Mean (US$)	9.23	4.86	1.82	4.94
Median (US$)	4.89	4.21	0.53	4.06
IQR	7.15	4.66	1.88	4.81
Central hospitals	Emergency cases (n = 25)	Elective cases (n = 61)		Total (N_2_ = 86)
Patients who incurred transport cost	17 (68%)	55 (90%)		72 (84%)
Mean (US$)	8.84	3.06		4.74
Median (US$)	2.56	1.88		1.81
IQR	11.28	2.48		3.31

At the district hospitals, which serve their patients just one very simple meal per day, most patients reported expenditure on food and groceries (98%), and about three-quarters had other types of expenditure (such as phone call charges; 77%; Table 2). At central hospitals the proportions were somewhat lower (87% and 73%, respectively). The median total expenditure by patients at district hospitals was higher than for patients at central hospitals (US$ 10.56 versus US$ 6.62, including cost of transport; p < 0.001).

**Table 2:** Expenditure incurred by patients due to surgery, by type of hospital (in US$).

		District hospital patients (N_1_ = 137)	Central hospital patients (N_2_ = 86)
Food & groceries	n	134 (98%)	75 (87%)
	mean	7.03	5.04
	median	4.96	3.34
Other expenditure	n	106 (77%)	63 (73%)
	mean	1.50	0.82
	median	0.75	0.38
Total expenditure, excl transport	n	134 (98%)	83 (97%)
	mean	8.53	5.86
	median	5.72	4.21
	IQR	6.77	5.23
Total expenditure, incl transport	n	136 (99%)	85 (99%)
	mean	13.47	10.60
	median	10.56	6.62
	IQR	11.81	7.33

Two-thirds (66%) of the patients treated at district hospitals reported loss of income (indirect costs) due to their admission, among whom the median income lost was US$ 90 (around MWK 60,000); with a mean loss of US$ 299 (five patients reported losses of more than US$ 1000 - data not shown in the table). This compared to less than a third (29%) of patients who underwent surgery at central hospitals who experienced income loss (p < 0.001; Table 3); whose median loss of income was US$ 71.5 (MWK 47,500), mean US$ 126.5. Likewise, loss of income by other household members (another form of indirect costs) was also reported more frequently by district hospital patients (26%, versus 8% of central hospital patients; p < 0.01).

**Table 3:** Income lost due to hospital admission for surgery (in US$).

	District hospital patients (N_1_ = 137)	Central hospital patients (N_2_ = 86)
Patients reporting loss of own income	91 (66%)	25 (29%)
Patients reporting loss of income by household members	36 (26%)	7 (8%)
Mean loss of income reported overall	213.53	38.25
Mean loss of income reported by those reporting income losses	299.38	126.50
Median loss of income reported overall	30.09	0.00
Median loss of income reported by those reporting income loss	90.27	71.46
IQR for those reporting income loss	203.10	161.73

Emergency patients at central hospitals spent more on transport than elective cases (median US$ 2.56 versus US$ 1.88; p < 0.05). For patients treated at district hospitals there was no difference in transport expenditure between emergency and elective cases, but this could be a chance finding due to few cases in the former category.

**Fig. 1:**
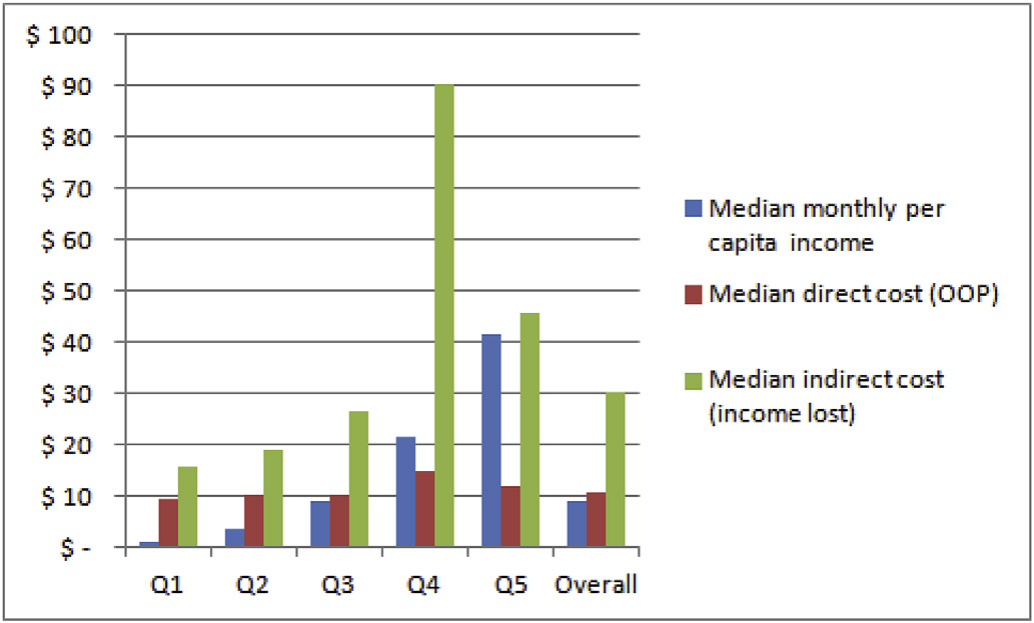
Median direct and indirect cost of accessing surgery at district hospitals (in US$) in relation to median monthly per capita income, by monthly per capita income quintile (N = 136).

Fig. 1, derived from Table A.6 in the Appendix, shows the median direct cost, paid by patients out-of-pocket (OOP) due to their admission to district hospitals, and the median indirect cost, indicated by their reported loss of income, by per capita income quintile. In the three poorest quintiles (Q1, Q2, Q3), the median direct cost of accessing surgery exceeds the median monthly per capita income. Households in Q1 paid almost 8 times their monthly per capita income to access surgery. This ratio is somewhat less dramatic in Q2 and Q3, at 2.7 and 1.2 respectively. Households in the two highest income quintiles (Q4 and Q5) spent on average less than their monthly per capita income to access surgery (ratios of 0.7 and 0.3 respectively). For only eight households in the total sample of 136 (6%) their expenditure did not exceed 10% of their monthly per capita income to access surgery, implying that all others (94%) incurred catastrophic expenditure (Table 4). When indirect costs, as indicated by reported loss of income, is taken into account, 97% of household incurred more than 10% of their monthly per capita income.

**Table 4:** Households that incurred catastrophic expenditure (direct cost >10% monthly per capita income) to access surgery and households for which the direct and indirect costs combined were more than 10% of their monthly per capita income.

	District hospital patients (N_1_ = 136)	Central hospital patients (N_2_ = 86)
Direct cost more than 10% of total monthly household income	100 (74%)	57 (66%)
Direct cost more than 10% of monthly per capita income a	128 (94%)	75 (87%)
Total cost (direct + indirect cost) more than 10% of total monthly household income	123 (90%)	65 (76%)
Total cost (direct + indirect cost) more than 10% of monthly per capita income	132 (97%)	77 (90%)

a World Bank definition of catastrophic health expenditure.

More than a third of the district hospital patients reported financial shortfalls (52 out of 136 patients, or 38%), meaning they were unable to pay for the directs cost out of their own pockets. The average reported shortfall was US$ 10.17 (sd = 11.53). Two-thirds of them borrowed money (34 out of 52; 65%). Others sold assets, received financial gifts or found other ways to cover the shortfall.

**Fig. 2:**
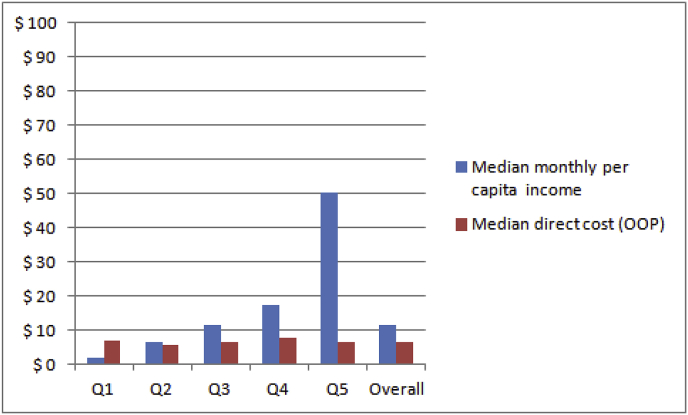
Median direct cost of accessing surgery at central hospitals (in US$) in relation to median monthly per capita income, by per capita income quintile (N = 86).

The pattern of expenditure associated with surgery by patients treated at central hospitals is shown in Fig. 2 (derived from Table A.7 in the Appendix). In Q1 the median direct cost of accessing surgery significantly exceeds the median monthly per capita income (by a factor 3.6). Households in Q2, Q3 and Q4 spent on average somewhat less than their monthly per capita income to access surgery (ratios of 0.9, 0.6 and 0.4, respectively). In Q5 (the highest quintile) the median direct cost was 13% of the median per capita income, with just 11 cases in the total sample of 86 (13%) whose direct expenditure did not exceed 10% of their monthly per capita income. This implies that all others (87%) incurred catastrophic expenditure (Table 4). When indirect costs, as indicated by reported loss of income, is taken into account, 90% of households incurred more than 10% of their monthly per capita income.

Almost a third of the patients managed at central hospitals (27; 31%) reported financial shortfalls, which were on average US$ 7.86 (sd = 8.08). Twenty-six patients (30% of the total) borrowed money; eight of them sold household assets (9%), seven received financial support (8%), and one received some other form of assistance.

Successful follow-up interviews by telephone were held with 66 patients (48%) who had their surgery at one of the district hospitals. Almost two-thirds (41 out of 66; 62%) had regained their capacity to work and generate income, although for three of them this was only partially the case as they were still hampered by abdominal pain. Twenty-five (38%) of them had not regained their capacity to work, still with considerable pain or not yet able to do any physical work. For some, the financial consequences were tough, as illustrated by the following response from one of the patients interviewed:

“I am unable to do my tomato business the way I used to do, as I cannot carry heavy baskets of tomatoes to the market.”

About a quarter of the re-interviewed patients (17; 26%) reported that their condition was still burdening them financially. About half (34 patients; 52%) indicated that their household's economic situation at the time of the follow-up interview had improved compared with the situation before they had their surgery. However, one fifth of the interviewees (14 patients; 21%) reported a deterioration of their economic situation, with a similar number reporting no change (13 patients; 20%).

## 4 Discussion

This is the first empirical study from Malawi that quantifies the financial burden of surgery from the perspective of households. It corroborates global estimates as well as estimates for Malawi of the probability of catastrophic expenditure if surgery is required [16], which were derived from a stochastic model. Empirical studies on household expenditure specifically for surgery were published recently from Bangladesh [23] and Uganda [[24], [25], [26]]. National patient-level data for out-of-pocket expenditure for surgery have been collected through patient exit interviews in Ghana, Kenya, Uganda, Zambia and India, by the Access, Bottlenecks, Costs and Equity (ABCE) project of the Institute of Health Metrics and Evaluation [27]. However, such data are needed from other settings, including Malawi. Our study informs two of the six global surgery key indicators proposed by the Lancet Commission on Global Surgery, i.e. Protection against impoverishing expenditure for surgical care and Protection against catastrophic expenditure for surgical care [3], and actually finds that the probability of people falling into poverty when they undergo surgery is somewhat higher.

Our study helps make the case for the pervasiveness of catastrophic health expenditure in low resource settings, observed by Njagi et al. [14], specifically in the case of general surgery, where the poor are the most affected. Surgical patients in Malawi are paying a catastrophic proportion of their monthly income out-of-pocket to access surgery. This study confirms that, even though government-owned hospitals in Malawi do not charge any fees for surgery or most other services that they provide, patients do incur substantial costs in accessing surgery, which in many cases exceed their monthly incomes, especially in the lower income quintiles. Unlike most other studies, we did measure opportunity costs due to income foregone because of hospital admission for surgery and found that such indirect costs often exceed the direct out-of-pocket expenditure. We found coping mechanisms, such as taking out loans or selling assets, fairly widespread, indicating an increased risk of further impoverishment.

Where this study breaks new ground is in reporting and comparing direct and indirect costs incurred by patients and their families in accessing the same type of surgery at district and central hospitals. The perhaps initially surprising finding of higher patient costs incurred at district compared to central hospitals can be explained. Reasons include the longer duration of stay for patients at district hospitals (median = 4.0) compared with central hospitals (median = 1.6), which would have had knock-on effects on their direct out-of-pocket payments, as well as on indirect costs, due to loss of income from time off work for patients and their families. Secondly, the impact of hernias and the recovery time from hernia repairs may have disproportionately impacted on rural patients, most of whom were farmers. Thirdly, lower transport costs for patients attending central hospitals, which is somewhat counterintuitive, may be because of their proximity to these hospitals. What this study could not measure was the comparative costs for individual patients of the alternative options of district or central hospital care. Hence the findings do not support the case for shifting hernia repair surgery from district to central hospitals; although it may well support the need to ensure district surgical clinicians have the skills to minimise patient inpatient stays.

### 4.1 Limitations

Our study has several limitations. First, the sample was not nationally representative as the study was carried out in six purposively selected health facilities (four district hospitals, two central hospitals). Also, since part of trade in Malawi is barter based, transactions cannot always easily be expressed in financial terms. Self-reported estimates of out-of-pocket expenditure, monthly household income and income forgone have their limitations too. Social desirability bias may have resulted in patients over-reporting expenditure, or giving partial information about their households’ sources of income and the amounts they earned in the month prior to their admission – which may have led to over- or underreporting. In addition, we do not know how much money patients would have spent anyway, had they not been admitted. Seasonal bias may have crept in, as especially rural household incomes fluctuate during the year. Furthermore, our estimates of indirect costs (lost income due to surgery) do not take into account that households reporting no or very little financial income, especially those that do not have anybody earning a steady wage, may actually lose opportunities to sustain themselves, by being unable for some time to grow their own food or earn themselves some money through casual labour. This implies that the actual indirect cost could be higher than our estimates. Lastly, although the two populations (district and central hospital surgical patients) were quite comparable in terms of total monthly household income, they were not in terms of monthly per capita income: central hospital patients were not as poor as district hospital patients because their households were smaller.

Despite these limitations, the presented data are potentially useful as complementary inputs to studies on the cost of surgery from a provider's perspective [28], one of which we conducted earlier in Malawi under the same project [29], and to feed into cost-effectiveness estimates of alternative models to perform surgery. Given the poverty levels in Malawi, it can be assumed that certain patients decide not to seek treatment for conditions that are amenable to surgery because of the cost involved. The exact scope and size of the unmet need for surgery in Malawi, as in most other SSA countries, is unknown [30]. It is clear though, that there is a need to protect those who can hardly or not afford the direct and indirect cost of accessing surgery. The provision of surgery by Government hospitals free of any charge to the patient is clearly insufficient. Meanwhile, surgical systems research should continue to inform the potential for scaling-up surgery at district hospitals, which are the first point of surgical care for rural communities, and support initiatives to actually scale-up safe surgery [30].

### 4.2 Conclusion

Out-of-pocket household expenditure associated with essential surgery in Malawi is high and in many instances catastrophic, putting households, especially those who are already poor, at risk of further impoverishment. The much needed scaling-up of surgical services in rural areas of Malawi needs to be accompanied by financial risk protection measures.

## Ethical Approval

The study was reviewed and approved by the University of Malawi College of Medicine Research Ethics Committee. Reference number P.03/12/1188.

## Sources of funding

The present manuscript is a product of the COST-Africa study, which was funded by the European Union’s 7^th^ Framework Programme for Research and Technological Development Grant, Ref:COST-AFRICA-2010, grant agreement no:266417. Salary support was funded by the European Union’s Horizon 2020 Programme for Research and Technological Development Grant, Ref: SURG-AFRICA-2016, grant agreement no:733391.

## Author contribution

LB, GM and DC conceptualised the study.

GM, DC: data curation.

LB, MW, DC, HB: formal analysis.

LB, DC, HB: methodology, including tools design.

GM, EB: project administration.

GM, DC: supervision of field work & validation.

MW, LB: writing of original draft manuscript.

JG, RB, EB, LB: review & editing of manuscript.

Contributors: none to be listed.

## Conflicts of interest

We declare no conflict of interest.

## Research Registration Number

The COST-Africa study was registered in the ISRCTN registry in February 2014, under number ISRCTN66099597

## Guarantor

Dr Leon Bijlmakers, Radboud UMC, The Netherlands

Prof Ruairí Brugha, RCSI, Ireland

## Provenance and peer review

Not commissioned, externally peer reviewed.

## Declaration of interests

None.
