# Supplementary material for: Out-of-pocket payments and catastrophic household expenditure to access essential surgery in Malawi - A cross-sectional patient survey
Source: Ann Med Surg (Lond). 2019 Jun 11;43:85–90. doi: 10.1016/j.amsu.2019.06.003 (PMC6580231; doi:10.1016/j.amsu.2019.06.003)
Supplement: Multimedia component 1 [file mmc1.docx]

Tables A.6 and A.7 to insert in Appendix of the Malawi household cost of surgery paper

**Table A.6** Direct and indirect cost for households to access surgery at district hospitals per household income quintile, in US$ (N=136*)

| Income quintile |  | Monthly household income | Monthly per capita income | Direct cost (OOP expenditure) | Indirect cost (income lost) | Total cost | Direct cost / monthly per capita income | Total cost /  monthly per  capita income |
| --- | --- | --- | --- | --- | --- | --- | --- | --- |
| Q1 (n=27), poorest | Mean  (sd) | 5.86  (4.35) | 0.99 | 11.61 | 75.86 | 87.47  (145.44) | 11.7 | 88.5 |
|  | Median | 7.52 | 1.20 | 9.24 | 15.80 | 25.65 | 7.7 | 21.3 |
| Q2 (n=27) | Mean  (sd) | 22.34  (6.79) | 3.91 | 11.08 | 76.32 | 87.40  (68.37) | 2.8 | 22.4 |
|  | Median | 22.57 | 3.76 | 10.34 | 19.18 | 31.59 | 2.7 | 8.4 |
| Q3 (n=28) | Mean  (sd) | 45.53  (11.33) | 9.47 | 15.06 | 308.92 | 323.98  (308.79) | 1.6 | 34.2 |
|  | Median | 45.13 | 9.03 | 10.46 | 26.33 | 44.56 | 1.2 | 4.9 |
| Q4 (n=27) | Mean  (sd) | 107.15  (29.16) | 21.21 | 16.22 | 348.71 | 364.93  (847.02) | 0.8 | 17.2 |
|  | Median | 105.31 | 21.40 | 14.67 | 90.27 | 96.36 | 0.7 | 4.5 |
| Q5 (n=27) | Mean  (sd) | 324.62  (254.41) | 60.53 | 12.70 | 254.29 | 266.99  (373.60) | 0.2 | 4.4 |
|  | Median | 223.41 | 41.52 | 11.73 | 45.89 | 54.76 | 0.3 | 1.3 |
| Overall (N=136) | Mean  (sd) | 100.69  (162.44) | 19.15 | 13.35 | 213.53 | 226,87  (719.76) | 0.7 | 11.8 |
|  | Median | 45.13 | 9.03 | 10.55 | 30.09 | 48.14 | 1.2 | 5.3 |

* One patient excluded from this analysis

**Table A.7** Direct and indirect cost for households to access surgery at central hospitals per household income quintile, in US$ (N=86)

| Income quintile |  | Monthly household income | Monthly per capita income | Direct cost (OOP expenditure) | Indirect cost (income lost) | Total cost | Direct cost / monthly per capita income | Total cost /  monthly per  capita income |
| --- | --- | --- | --- | --- | --- | --- | --- | --- |
| Q1 (n=17), poorest | Mean  (sd) | 7.54  (6.04) | 1.85 | 11.95 | 67.26 | 79.20  (148.03) | 6.5 | 42.9 |
|  | Median | 7.52 | 1.88 | 6.77 | 0.00 | 6.92 | 3.6 | 3.7 |
| Q2 (n=17) | Mean  (sd) | 27.74  (5.29) | 6.57 | 7.96 | 29.65 | 37.61  (23.77) | 1.2 | 5.7 |
|  | Median | 30.09 | 6.39 | 5.48 | 0.00 | 9.33 | 0.9 | 1.5 |
| Q3 (n=18) | Mean  (sd) | 44.92  (5.01) | 11.72 | 10.42 | 34.37 | 44.79  (86.44) | 0.9 | 3.8 |
|  | Median | 45.13 | 11.41 | 6.62 | 0.00 | 11.21 | 0.6 | 1.0 |
| Q4 (n=17) | Mean  (sd) | 73.77  (14.22) | 18.92 | 15.08 | 16.37 | 31.46  (106.42) | 0.8 | 1.7 |
|  | Median | 72.21 | 17.55 | 7.82 | 0.00 | 9.03 | 0.4 | 0.5 |
| Q5 (n=17) | Mean  (sd) | 355.49  (695.83) | 139.88 | 7.61 | 43.81 | 51.42  (94.10) | 0.1 | 0.4 |
|  | Median | 133.89 | 50.15 | 6.47 | 0.00 | 7.97 | 0.1 | 0.2 |
| Overall (N=86) | Mean  (sd) | 101.23 (328.30) | 35.51 | 10.60 | 38.25 | 48.85  (100.06) | 0.3 | 1.4 |
|  | Median | 45.13 | 11.41 | 6.62 | 0.00 | 8.58 | 0.6 | 0.8 |
