# Supplementary material for: Out-of-pocket payments and catastrophic household expenditure to access essential surgery in Malawi - A cross-sectional patient survey
Source: Ann Med Surg (Lond). 2019 Jun 11;43:85–90. doi: 10.1016/j.amsu.2019.06.003 (PMC6580231; doi:10.1016/j.amsu.2019.06.003)
Supplement: Multimedia component 2 [file mmc2.doc]

**International Journal of Surgery Author Disclosure Form**

Completed for the manuscript entitled ‘Out-of-pocket payments and catastrophic household expenditure to access essential surgery in Malawi’.

16 March 2019

The following additional information is required for submission. Please note that failure to respond to these questions/statements will mean your submission will be returned. If you have nothing to declare in any of these categories then this should be stated.

**Please state any conflicts of interest**

| We declare no conflict of interest. |
| --- |

**Please state any sources of funding for your research**

| The present manuscript is a product of the COST-Africa study, which was funded by the European Union’s 7^th^ Framework Programme for Research and Technological Development Grant, Ref:COST-AFRICA-2010, grant agreement no:266417. Salary support was funded by the European Union’s Horizon 2020 Programme for Research and Technological Development Grant, Ref: SURG-AFRICA-2016, grant agreement no:733391. |
| --- |

**Please state whether Ethical Approval was given, by whom and the relevant Judgement’s reference number**

| The study was reviewed and approved by the University of Malawi College of Medicine Research Ethics Committee. Reference number P.03/12/1188. |
| --- |

**Research Registration Unique Identifying Number (UIN**)

Please enter the name of the registry and the unique identifying number of the study. You can register your research at [http://www.researchregistry.com](http://www.researchregistry.com/) to obtain your UIN if you have not already registered your study. This is mandatory for human studies only.

| The COST-Africa study was registered in the ISRCTN registry in February 2014, under number ISRCTN66099597 |
| --- |

**Author contribution**

Please specify the contribution of each author to the paper, e.g. study design, data collections, data analysis, writing. Others, who have contributed in other ways should be listed as contributors.

| Authors:  LB, GM and DC conceptualised the study.  GM, DC: data curation.  LB, MW, DC, HB: formal analysis.  LB, DC, HB: methodology, including tools design.  GM, EB: project administration.  GM, DC: supervision of field work & validation.  MW, LB: writing of original draft manuscript.  JG, RB, EB, LB: review & editing of manuscript.  Contributors: none to be listed. |
| --- |

**Guarantor**

The Guarantor is the one or more people who accept full responsibility for the work and/or the conduct of the study, had access to the data, and controlled the decision to publish.

| Dr Leon Bijlmakers, Radboud UMC, The Netherlands  Prof Ruairí Brugha, RCSI, Ireland |
| --- |
